# Refugee Policy Implications of U.S. Immigration Medical Screenings: A New Era of Inadmissibility on Health-Related Grounds

**DOI:** 10.3390/ijerph14101107

**Published:** 2017-09-24

**Authors:** Mi-Kyung Hong, Reshma E. Varghese, Charulata Jindal, Jimmy T. Efird

**Affiliations:** 1 Centre for Clinical Epidemiology and Biostatistics (CCEB), School of Medicine and Public Health, The University of Newcastle (UoN), Callaghan, NSW 2308, Australia; hong.mkhong@gmail.com (M.-K.H.); charulata.jindal@newcastle.edu.au (C.J.); 2 School of Medicine and Health Sciences, George Washington University, Washington, DC 20037, USA; reshmaelsamvarghese@gmail.com; 3 Center for Health Disparities (CHD), Brody School of Medicine, East Carolina University (ECU), Greenville, NC 27834, USA

**Keywords:** HIV, immigration, measles, populism, refugee health, rule-making, tuberculosis

## Abstract

Refugees frequently face extended delays in their efforts to enter the United States (U.S.) and those who are successful, in many cases, encounter overwhelming obstacles, inadequate resources, and a complex system of legal barriers. Travel restrictions based on equivocal health concerns and a drop in refugee admittance ceilings have complicated the situation. The authors retrieved and analyzed peer-reviewed journal articles, government agency press releases, media postings, epidemiologic factsheets, and relevant lay publications to critically assess U.S. policy regarding refugee resettlement based on health-related grounds. While refugees arguably exhibit an increased incidence of measles and tuberculosis compared with the U.S. population, the legitimacy of the medical examination will be undermined if other diseases that are endemic to refugee populations, yet currently deemed admissible, are used to restrict refugees from entering the U.S. This paper addressees the historic refugee policy of the U.S. and its consequent effect on the health of this vulnerable population. The needs of refugees should be carefully considered in the context of increased disease burden and the associated health care challenges of the country as a whole.

## 1. Introduction

Refugee health is a complex and political issue in terms of risk preparedness, resource management, surveillance, and overall management. The situation is confounded by the multicultural and multilingual diversity of this population and their different needs in comparison to their host country. Additionally, health-related and security-related grounds for admissibility and the entry of refugees into the United States (U.S.) often has been influenced by populism and partisan ideology rather than fact-based public health evidence [1,2,3]. Below, we provide an overview of refugee policy in the United States (U.S.) and its implications on preparedness efforts and crisis management.

## 2. Literature Review

### 2.1. History and Background

We analyzed historic U.S. refugee policy and measures taken by governmental and non-governmental organizations that have been instrumental in deciding the medical screening of refugees entering the U.S. Information was retrieved from peer-reviewed journal articles, government agency press releases, media postings, epidemiologic factsheets (from public health organizations) and relevant lay publications to critically assess U.S. policy regarding refugee resettlement based on health-related grounds.

#### 1980 Refugee Act

Refugee case management and policy in the U.S. continues to be a perplexing and complex process. To better understand the limitations of migrant and refugee health in the context of this discussion, we provide a summary of the U.S. refugee act of 1980. This landmark act provides comprehensive legislation granting government bodies authority over decisions needed to guide refugee admittance to the U.S. [4]. U.S. immigration legislation that oversees and directs immigration and citizenship policy is codified in the Immigration and Nationality Act (INA). The INA designates refugee status to those individuals who are unable to return to their native countries owing to race, religion, nationality, or membership in a certain social group, or of a political opinion [4].

The Refugee Act is primarily concerned with three objectives in its statutory jurisdiction. These objectives are (1) to provide humanitarian response that is expedient to the arising crises; (2) to act as key responders with the flexibility to engage in such endeavors, whereby ad hoc activities are to be mitigated; and (3) to govern the process of refugee admittance to the U.S. with less emphasis placed on geographic resident concerns [4]. The President enumerates the yearly number of refugees allowed admittance to the U.S., known as the refugee ceiling, after consultation with Congress [5]. For the year of 2015, the U.S. refugee ceiling of admissible refugees worldwide was 70,000 [5]. Allocations for 2015 were as follows: African (17,000), East Asia (13,000), Europe and Central Asia (1000), Latin America/Carribean (4000), and Near East/South Asia (33,000). A pool of 2000 admissible refugee determinations are left unallocated to accommodate new circumstances requiring U.S. assistance [5].

Priorities for U.S. foreign policy that affect the refugee ceiling fall into 3 categories. Firstly, an individual’s circumstances must have security concerns such that it is paramount that they are granted asylum. Secondly, the U.S. gives special humanitarian status to specific refugee populations such that they are prioritized. Thirdly, preserving family unity of refugees already granted asylum in the U.S. is a directive [5]. Often, the U.S. identifies certain refugees to be of “special concern”. This vague terminology is prevalent and we present clarification of this statutory language. U.S. foreign policy agendas, individuals with previous ties to the U.S. in some fashion, and family reunification are primary qualifications for those designated as a “special concern” [4]. The U.S. accepts refugees from overseas and the refugee admittance process begins in their native countries or an asylum country.

In the U.S., agencies that oversee refugee admission are the Department of State (DOS) and the Department of Homeland Security (DHS) [6]. Each have a specific and defined role. For example, DOS manages administrative processes such as issuing visas. The eligibility for U.S. residency of incoming migrants is under the auspices of the U.S. Citizenship and Immigration Services (USCIS), a subagency of DHS. The USCIS confirms the ultimate determination about refugee status and whether admittance is possible for those seeking such status [6]. Of significant concern for refugee communities and their admissibility to the U.S. is their status in relation to health-related and security-related grounds of inadmissibility. The two agencies that adjudicate and oversee this process are the Department of Health and Human Services (HHS) and the Centers for Disease Control and Prevention (CDC), a subagency of HHS [6].

Communicable diseases are fundamental in the determination of inadmissibility on health-related grounds to the U.S. Our focus is to discuss and analyze the complexities of health-related grounds of inadmissibility, with special attention to communicable diseases. Pertinent to our discussion, the HHS and the CDC are the agencies of most relevance to refugee health status. The Secretary of HHS identifies communicable diseases that can threaten U.S. public health and he/she dictates disease inadmissibility on health-related grounds [6]. The CDC authoritatively determines what public health measures need to be addressed regarding foreign nationals in order to protect the health of the American public [6].

### 2.2. The Medical Exam

The INA requires all refugees applying for U.S. immigration to receive medical screenings for inadmissible health conditions [7]. Refugees undergo overseas medical examination prior to departure to the U.S., a consequent U.S.-based medical examination, and a repeat examination when applying for adjustment of their immigration status to that of a permanent resident [8,9]. The three primary medical screenings are outlined below.

#### 2.2.1. Overseas Medical Examination

A medical examination is required for all immigrants and refugees entering the U.S. Approximately 400–800 panel physicians are authorized by U.S. embassies and consulates oversees to conduct the medical examinations. The examination identifies if the applicant has a physical or mental condition that renders them inadmissible for a visa (Class A condition), or if they have a physical or mental condition that, although does not render them inadmissible to the U.S., is significant enough to interfere with the applicant’s ability to take care of themselves, or attend school or work, and may require extensive medical treatment or institutionalization (Class B condition). The medical examination involves review of medical history, review of records that may help determine history of harmful behavior related to health condition, review of symptoms, and a physical examination [8].

#### 2.2.2. U.S. Domestic Medical Examination for Newly Arriving Refugees

Usually within 30–90 days post arrival in the U.S., refugees undergo a domestic medical examination [7]. The CDC has published recommendations to assist public health departments and medical professionals to identify appropriate tests to perform during refugee medical screening.

#### 2.2.3. Medical Examination for Adjustment of Status

One year after being admitted into the U.S., refugees are required to apply for a Green Card to receive permanent U.S. residence. A medical examination performed by a U.S. physician authorized as a civil surgeon is required for adjustment of status to permanent U.S. resident. A civil surgeon must complete parts of the Report of Medical Examination and Vaccination Record (Form I-693) regarding vaccination requirements at the time of adjustment of status for all refugees. However, only refugees with a Class A medical condition identified during the overseas medical examination need to undergo the entire medical examination. Asylees, on the other hand, are required to undergo a medical examination and vaccination assessment during adjustment of status. An immigration officer can require a refugee/asylee applicant to undergo a medical examination at any time if there is evidence of a public health concern [9]. Form I-693 also includes assessment of drug addiction/abuse, tuberculosis (TB), syphilis, gonorrhea, Hansen’s disease, and mental illness associated with harmful behavior [10].

### 2.3. Health-Related Grounds for Inadmissibility

The overseas medical examination and the medical examination for adjustment of status identifies health conditions that are grounds for inadmissibility into the U.S. [7]. Applicants can be found inadmissible based on the following health grounds:

#### 2.3.1. Communicable Diseases

Applicants who are diagnosed with communicable diseases of public health significance, also known as Class A conditions, are inadmissible. Class A conditions include gonorrhea, leprosy (infectious), syphilis (infectious), and TB (active) [11]. Latent TB infection (LTBI), syphilis, and gonorrhea that were treated within the past year, and treated or partially treated Hansen’s disease are collectively classified as Class B conditions and do not impact the applicant’s admissibility. Applicants are tested for most of these conditions only if they meet certain age requirements [10]. Another category for communicable diseases of public health significance are diseases that may subject the applicant to federal isolation and quarantine. These diseases include cholera, diphtheria, infectious TB, plague, smallpox, yellow fever, viral hemorrhagic fevers, severe acute respiratory syndromes, flu that can cause a pandemic, and other diseases that may pose a public health emergency of international concern [12]. However, immigration officers are advised that these conditions are only applicable to the applicants abroad [11].

#### 2.3.2. Physical or Mental Disorder with Associated Harmful Behavior

Applicants who are diagnosed with physical or mental disorders and associated harmful behaviors can be identified as inadmissible. This may apply to current disorders with associated harmful behaviors, or past disorders with associated harmful behaviors that have a tendency to either recur or develop into additional harmful behaviors. It is important that the applicant has both a disorder and a harmful behavior in order to be denied adjustment of status [13].

#### 2.3.3. Drug Abuse or Drug Addiction

Applicants who are diagnosed with substance use disorders are rendered as inadmissible. However, the applicants are able to reapply for adjustment of status if they are in remission [11].

#### 2.3.4. Vaccination Requirement

Applicants are required to meet certain vaccination requirements in order to successfully adjust their status. If the applicant has not received vaccinations mandated by the INA and CDC, and the vaccinations are age and medically appropriated, he/she is deemed to have a Class A condition and is inadmissible based on health grounds. The vaccinations mandated by INA and CDC are mumps, measles, rubella, polio, tetanus and diphtheria toxoids, pertussis, haemophilius influenza type B, hepatitis B, varicella, influenza, pneumococcal pneumonia, rotavirus, hepatitis A, and meningococcal [14].

#### 2.3.5. Waivers

If the applicant has a communicable disease of public health significance or a medical disorder that is classified as a Class A condition, he/she may be eligible for certain waivers under INA. Waivers are available for communicable diseases of public health significance, physical or mental disorders, and unmet vaccination requirements [15]. Once the USCIS deems an applicant eligible for a waiver, applications are reviewed by HSS and CDC. CDC determines if the applicant is correctly examined, diagnosed, and classified. Although USCIS ultimately approves the waiver, CDC’s assessment strongly influences the conditions associated with the waiver and if USCIS grants the applicant a waiver [16].

### 2.4. Historical Changes in the Medical Exam: The Human Immunodeficiency Virus (HIV) Ban

A significant component of the U.S. medical examination for immigration is an individual’s requisite absence of any communicable diseases of public health significance. The regulation of HIV infection among foreign nationals upon U.S. entry was historically determined by the list of communicable diseases of public health significance. [17] This adjudication spanned the course of 22 years in the 20th and 21st century [17]. HIV was designated on the list as a paramount contagious illness that posed harm to the American people if contact ensued with foreign nationals. Statutory power promulgated HIV to be a significant threat to the U.S. public and a disease of public health significance. Where once U.S. law created the HIV ban, statutory powers eliminated HIV from the U.S. medical examination list of contagious illnesses in 2010 [18].

The HIV ban was promoted for two essentials reasons. First, as a public health measure it ensured that foreign nationals would not expose U.S. people to a fatal infectious disease [3]. Second, the U.S. would not inadvertently, by admitting those infected with HIV, become a host country subject to “public criticism” and misdirected national resources [3]. The origin of the HIV ban began in 1987 when acquired immune deficiency syndrome (AIDS) was initially added to the list of communicable diseases of public health significance by the Department of Health and Human Services (DHHS) [3]. However, shortly thereafter, the ban included all individuals with HIV infection and not solely those who had advanced to AIDS. The HIV ban was sustained as status quo for 22 years as legislative bodies debated the risk level for this disease [18].

Although the ban affected all ethnic and racial groups afflicted with HIV infection, a disproportionate amount of the burden was borne by non-white populations in underdeveloped countries. One such immigrant group, Haitians, experienced discriminatory behavior in the HIV ban era on the part of panel physicians, civil surgeons, and U.S. immigration officials [19]. This is “Katiana’s” Story [19]:

“I am Haitian and my brothers, mother and stepfather are all in the U.S. I didn’t know anything about HIV until I was forced to get tested as part of the visa application process. When I found out I had HIV, I was afraid to tell my family ... I found out [about] the HIV waiver ... Officials at the Embassy are rude to people with HIV, and anybody who works at the Embassy knows that you have HIV/AIDS because there are only certain days people with health problems can come to the Embassy. Even on those days, there are special lines inside the Embassy for people with HIV/AIDS.”

A central principle of those who opposed the HIV ban at its inception and throughout its course was the “presumed” legitimacy of such a ban. The historical medical facts surrounding the AIDS epidemic showed that at the time of the HIV ban’s origin the largest count of HIV/AIDS cases was in the U.S. [20]. Also, knowledge of HIV infection and its spread did not warrant the ban as HIV is not casually transmitted. The potential civil liberties breach, such as the breach of confidentiality of HIV infection [19] created by the HIV ban, was troublesome to many and showed a contradiction in U.S. policymaking [20]. As AIDS research and treatment advanced in the 1990s and into the millennium, HIV/AIDS became a disease burden that was manageable with the advent of combination antiretroviral therapy. Fatality was not the immediate endpoint and case fatality saw a vast reduction. However, policy surrounding HIV did not reflect current knowledge about this disease [18]. Yet, in due course, no defensible support existed for the HIV ban. The medical advances made in the U.S., the geopolitical advocacy engagement, and the civic leadership that the U.S. government held in AIDS policymaking created a contrary ethos to the HIV ban [3,18]. For instance, the targeting of non-White populations from low income countries, such as Haiti, has specifically raised concerns of racial prejudices and the profiling of HIV patients.

The Center for Strategic and International Studies (CSIS), an advisory committee to the U.S. Congress, became central in dismantling the HIV ban [3]. A domestic source of oversight expertise, CSIS published in March 2007 a report on immigration and HIV/AIDS that directly stated the untenable position the U.S. held in maintaining the HIV ban [3]. The CSIS report was instrumental in changing HIV legislation. Congressional leaders in July 2008 motioned to remove the language “which shall include infection with the etiologic agent for AIDS” from the INA [3]. In so doing, DHHS had independent jurisdiction to determine the list of communicable diseases of public health significance [17]. In 2009, the DHHS introduced a rule to remove HIV from the list of inadmissible communicable diseases [18]. During the public commentary phase of rule-making, 19,500 comments out of 20,100 submitted comments addressing HIV status inadmissibility supporting the new rule-making [18]. Successful elimination of the HIV ban ensued thereafter with rule-making enacted on 4 January 2010 [21].

### 2.5. Risk Communication

Health risks are inherently significant not only owing to their purported impact on a susceptible population but also because information and bias can pose a quandary. Risk communication is central to achieving public health security at the time of disease spread but it is also important in disease prevention and management [22,23]. A useful summation of risk communication goals is “to provide the public with meaningful, relevant, accurate and timely information in relation to health risks in order to guide decision making [22]”.

#### 2.5.1. Perception of Disease Migration and Refugee Displacement

Communicable diseases are by nature intricate phenomena with evolving and newly emerging strains of viruses. There is also the complexity of reintroduction of communicable disease in a disease-eradicated community, which can occur with a new influx of refugee migrants. Furthermore, environmental infrastructure and lifestyle play an important role in disease transmission [24]. Science is often biased by perception, created in part by media and other channels of risk communication. Factors associated with a population’s perception of disease risk may unduly influence legislation. This is the dilemma facing administration and health officials in their efforts to best carry out the duties of refugee resettlement. Factual science often cannot counter uncertain public opinion about the health risks posed by refugee resettlement.

There is a premise that migrants and refugees have an acquired infectious disease rate higher than that of host populations in developed countries, such as in Europe. This is debatable with many factual sources contradicting this assertion [24]. This conception challenges government programs accepting refugees for resettlement, as refugee adoption may cause potential burden on contentious societal resources [25]. Their placement also must appeal to humanitarian views and thus the perception of disease risk is a critical juncture for policy and practice.

With recent acts of terrorism in high-income countries that resettle refugees, an association of a security threat is also prevalent [24,26]. This phenomenon will no doubt aggravate public perception of disease risk that will be seen in tandem with national security and refugee migration. Risk communication will often be mischaracterized and scientific evidence may not advance the agenda to educate, defeating resettlement policy programming [27]. Prejudicial reasoning may prevail and public health officials must counter such beliefs with proper evidence of true disease risk. The overall perception of insecure access to care, and uncontrolled violence in refugee countries of origin impedes resettlement efforts.

Despite rigorous epidemiologic data to the contrary, rhetoric can influence the public with its populist vision. Government officials can relay the facts but press and social media frequently characterizes risk communication with the target audience being the lay population [22,23]. In such a climate, politicians must elicit “buy-in” from their constituents by opposing refugee resettlement.

#### 2.5.2. Risk Communication in the U.S.: Populist Case Studies

Below we present the complexity of risk perception of refugee disease and provide case studies published in the socially conservative media. Such articles highlight the pitfalls of risk communication.

##### Case Study: “Public Health Officials Silent on Possible Connection between Measles Outbreak and Resettled Refugees” 12 May, 2016 [28]

This news article reports a measles outbreak at the Masjid Al-Noor Mosque in Memphis, Tennessee and the pursuant commentaries from government officials and editorial remarks.

“The lack of immunization requirements for refugees entering the United States is deeply troubling to me. For months now, I have called on the federal government to halt our refugee resettlement program in its entirety and voted against last December’s omnibus bill because it failed to make these needed reforms. As we look ahead to this year’s funding bills, I am proud to have signed a letter to the House Appropriations Committee asking that the Fiscal Year (FY) 2017 Homeland Security appropriations bill force tighter controls on our refugee resettlement program. Open borders and unverified refugees from radical Islamic hotspots are a threat to our national security, our public health, and our very way of life.”

“The Office of Refugee Resettlement is but one of many federal departments that not only needs to be completely defunded but abolished. As with the illegal immigration problem, Congress has repeatedly reauthorized and expanded [the President’s] initiative to bring Muslim Refugees from radicalized Islamic countries into Tennessee”.

“We (news outlet) asked why state and local health officials have not taken the next obvious investigative step: to identify the immigration and vaccination status of the individual who was at the Masjid Al-Noor Mosque on 15 April—most likely an adult Muslim—who was, if not the first person infected with measles, then was one of the first...”

##### Case Study: “Health Expert Blasts CDC: Ignores own study, allows refugees into U.S. without latent TB screening. May 20. 2016”[29]

This news article criticizes the CDC for allowing refugees to enter the U.S. without screening.

“Admitting people who might cause an epidemic makes no sense whatsoever from a public health standpoint … It suggests that those who favor it do not care about the cost in suffering, death and expense to Americans.”

“If for humanitarian reasons we wish to help people fleeing persecution, there is still no need to release them into the general population of susceptible individuals. Officials who place politics above the health of Americans need to be held accountable and removed from positions of authority.”

Currently, however, the CDC does not screen or test the 70,000 refugees brought into the U.S. annually under the federal refugee resettlement program for LTBI. Refugees are tested for active TB and allowed entry into the U.S. subsequent to what the CDC determines is successful treatment. In 2014, among persons of known national origin, 66.5% of all active TB cases in the United States were among foreign-born persons, and the case rate among foreign-born persons was 13.4 times higher than among U.S.-born persons (15.3 versus 1.1 cases per 100,000 persons).

### 2.6. TB Infection and LTBI: Factual Risk

While media reports have heightened public concern, TB is factually among the top ten diseases that cause the highest mortality rates worldwide [30]. Although preventable and curable, monitoring and treatment efforts have been suboptimal in developing countries [30,31]. The disease is caused by *mycobacterium tuberculosis*, a bacteria which impacts mostly lung function [30]. Prior to 2011, more than 80% of all cases of TB in the U.S. were caused by LTBI reactivation to active TB disease [32]. Foreign nationals develop TB infection at rates of infection seen in their countries of origin owing to reactivation of LTBI [33]. Moreover, refugees experience higher rates of TB disease after U.S. resettlement than those seen among other foreign-born populations in the U.S. [33].

Approximately, one-third of the world’s population has LTBI [30]. This poses indeterminate risk to public health in the U.S., as administrative practices related to TB and LTBI screening are culpable for disease spread. Immigration policy and the adjudication of the U.S. medical examination cause gaps in monitoring TB and LTBI. The time period overlapping the administration of the overseas medical examination, whereby a refugee shows no signs of active TB, to when he/she enters the U.S., may be lengthy. During this period, an individual’s LTBI may reactivate to TB disease or it may do so shortly after U.S. resettlement [33,34]. With the ease of TB disease spread by respiratory routes, reactivated LTBI becomes a pernicious threat. Given such a scenario for potential public harm, LTBI, which is admissible, may become an inadmissible disease changed by government rule-making.

### 2.7. Measles: Factual Risk

The risk posed by measles imported from abroad, whether from migrant movements or from international travelers in transit, is an alarming circumstance for U.S. health officials. Measles is severely infectious and can become acute, which for individuals affected results in rash, respiratory symptoms, and fever [35]. With cases that become overwhelming, death results from complications such as pneumonia and encephalitis [35]. The concern for U.S. health officials regarding importation and outbreaks of measles in the U.S. is attributable to the status of measles epidemiology currently in the country. Past vaccination campaigns, which targeted children and included surveillance systems for detecting cases to prevent outbreaks, achieved elimination of the disease in the U.S. The World Health Organization (WHO) declared such elimination status in 2000 as measles was no longer deemed endemic in the U.S. [36].

Nevertheless, there are challenges to maintaining measles elimination in the U.S. The high volume of travelers that come to the U.S. yearly and the susceptibility of infection for those unvaccinated residing in the U.S. owing to personal creed create a margin of error [35]. A recent study showed that a large number of U.S. measles cases after the status of elimination in 2000 were due to those who intentionally remained unvaccinated [37]. This study revealed that unvaccinated children who intentionally opted out of receiving the vaccine were 35 times more likely to acquire measles compared with those vaccinated [37]. Such epidemiological data, though compelling, often cannot sway personal belief systems or change misinformed opinion. Resistance to health education and consequent importation of disease remains a reality.

Vaccine-preventable diseases such as measles and U.S. inadmissibility have become politically sensitive issues, especially for immigration policy. Codification in the U.S. medical examination allows refugees to enter the U.S. unvaccinated and refugees are given a grace period after entry to receive required vaccinations [7]. Refugees are the only group of immigrants given such an exemption.

An admissible condition for refugees such as unvaccinated status can become a controversial policy issue. This is particularly the case given successful efforts by the U.S. to eliminate measles, as balanced against the possibility of imported measles cases reintroducing the disease. The measles incubation period, from exposure to symptoms of fever, is 7 to 21 days. On the other hand, the characteristic rash can be seen 14 days after exposure to the virus. Infected individuals typically are contagious from 4 days before to 4 days after appearance of the rash. This allows for a 22 day period when the individual is contagious [38]. The possibility of acquiring a vaccine-preventable illness in the above context warrants public health intervention. Displaced individuals abroad are at a high risk of measles infection owing to massive displacement events, population density, and overcrowding at camps [39,40]. The spread of measles and outbreaks also occur because of the large number of unvaccinated children in refugee camps [40]. The WHO declares refugees as a high-risk population susceptible to measles outbreaks [40]. Contributing to the concern, public health measures, such as vaccination programs, are difficult to implement in refugee camp conditions [39,41]. Avoidable disease is thus common and indicates the lack of stability of the existent health systems caring for refugees [41]. Given these barriers, U.S. refugee policy recognizes the difficulty that this group has in receiving appropriate immunization.

In 2014, the CDC reported that a record number of measles cases had occurred with 667 cases originating from 27 states [42]. This level of measles cases in U.S. has not been observed since the declared elimination of the disease in 2000 [42]. However, this rise is primarily attributed to foreign travelers rather than the influx of immigrants and refugees [42]. From 2005–2008, the largest source of measles cases into the U.S. was from the WHO European Region (England, France, Germany and India) [7,35]. To date, there is little epidemiologic evidence to suggest that the immigrant and refugee population significantly contribute to the health risk of measles in the U.S.

### 2.8. Consequential Risk Communication from Expert and Non-Expert Debate: Federal Rule-Making

Effective governance deems that policy decision-making should be grounded on an evidential foundation. However, the interface between influential constituents and politicians has the potential to bias rule-making, especially when misinformed by reactionary (ultra-right) members of the lay press. Just as political pressure aided changes in rule-making with the HIV ban, the U.S. may face similarly contentious rule-making with respect to the health of immigrants and refugees.

#### 2.8.1. Rule-Making in the United States

While the U.S. Congress passes statutory law, such legislation requires detailed rule-making to pass into effect [43]. Rule-making agencies must be answerable to the studies and factual evidence in the field that inform any proposed regulation [43]. The explanation documenting new rule-making is titled the notice of proposed rule-making (NPRM). NPRM is an influential document and is managed and reviewed by the White House Office of Management and Budget (OMB) [43]. In the routine channels of OMB, NPRM is evaluated to determine if it is a match for presidential policy [43]. Public commentary plays an inherent role in the process of definitive rule-making and contributes to the designation of NPRM as legal regulation. A public citizen may repudiate new rule-making if he/she believes it is illegitimate and incoherent with Congressional statute [43]. A citizen can also oppose rule-making if he/she believes it neglects important input from commentary, or if he/she finds rule-making grossly remiss. Rule-making may also be challenged in U.S. courts [43].

#### 2.8.2. Executive Administration and Rule-Making

Agencies may change and reverse existing regulations by addressing their reasoning and by responding to changes in factual definitions that supported the prior policy [44,45]. Within these circumstantial limits, the President can revoke many existing regulations [45]. Public engagement may delay or hinder executive directives. U.S. citizens can also litigate to challenge new rule-making. It is unclear how judicial review will treat an agency’s rule-making governed by the White House. U.S. courts will apply the lesser “arbitrary and capricious” review standard that gives deference to the agency’s findings [45].

### 2.9. Current Developments Regarding Immigration Health

Two recent Presidential executive orders have outlined travel bans for refugees and immigrants from certain countries. In addition to restricting entry of immigrants from Iran, Iraq, Libya, Somalia, Sudan, Syria, Yemen, and possibly other countries, the executive order signed 27 January 2017 imposed significant changes on the refugee program. These changes included (1) suspending the refugee resettlement program for 120 days; (2) prioritizing refugee claims of religious minorities; (3) indefinitely suspending entry of refugees from Syria; (4) decreasing the refugee admission ceiling for FY 2017 from 110,000 to 50,000; and (5) allowing state and local authorities to be granted a greater role in determining if refugees are able to resettle in their jurisdictions. A revised executive order was signed on 6 March 2017. Similar to the previous order, the new order suspends travel of refugees into the U.S. to 120 days, decreases the refugee admission ceiling in FY 2017 to 50,000, and allows state and local authorities to play a greater role in determining eligibility of refugee resettlement in their jurisdictions. The new order, however, only suspended entry of Syrian refugees for 120 days and did not prioritize refugee claims of religious minorities [46]. Parts of both orders were temporarily halted owing to legal challenges.

The above-mentioned executive orders highlight an agenda of targeting refugees and certain immigrants as a threat to the security and welfare of the U.S. Refugees admitted to the U.S. currently undergo a thorough vetting process that can last from 9 to 24 months. Based on the applicant’s medical conditions, medical examinations are only valid between 3 to 6 months [47]. If an applicant’s screening expires during the 120-day suspension period under the executive order, they will have to undergo security checks and medical examinations again [48]. Although the executive order lists a 120-day refugee admission suspension, its impact on upstream vetting processes can delay refugee admissions for longer than the 4 month period listed in the executive order.

The history of American immigration depicts the strong role of medical screenings in anti-immigration policies. As early as 1910, the Federal government authorized local officials to govern medical screenings of immigrants [49]. On the West Coast, the Federal government established the Angel Island immigration station and hospital to govern medical and psychological examinations for U.S. bound immigrants [50]. If they did not pass the medical criteria, they were detained at length (sometimes for years) from entering the U.S. If individuals were not cured after treatment, deportation was recommended. Furthermore, immigrants who arrived on first or second class tickets were not subjected to the extensive medical examinations those in steerage had to undergo. From 1910 through 1940, individuals immigrating to the U.S. West Coast, and that were subsequently subjected to such health screenings and perhaps racial profiling, were primarily from Asia [50].

The immigration practices of the early 1900s highlight the association of disease with certain racial and class stereotypes. Even after over a century, the belief that infectious diseases originate abroad and are imported into the U.S. by immigrants still prevails. This perception of risk is reinforced by policies that mandate medical examinations from refugees and immigrants, and not from individuals visiting the U.S. or U.S. citizens returning from abroad. The HIV ban that extended from 1987 through 2010, despite the scientific confirmation of HIV infection’s restricted mode of transmission, accentuates how policies are employed to restrict refugee and immigrant entry and create a false perception that immigrants are at higher risk of transmitting disease to the U.S. population [51]. The recent executive orders share a similar sentiment of targeting immigrants despite opposing reports regarding banned immigrants’ increased risk to U.S. security [52].

## 3. Conclusions

The U.S. has traditionally played a leading role in providing humanitarian assistance to a world in crisis. Recognizing the needs of refugees is consistent with our natural heritage and belief that all people deserve equitable access to resources. Restricting admissibility on health grounds for diseases such as HIV, TB, and measles can have a detrimental impact on refugees and the general U.S. population. These measures can contribute to stigma and discrimination and inhibit refugees living in the U.S. from seeking treatment. Such policy can promulgate the misperception that disease burden is predominantly of foreign origin and can only be managed through border controls rather than treatment, public health education, and other preventative methods [53].

The U.S. Refugee Act of 1980 established the Federal Refugee Resettlement Program, which allowed for the admission of 3 million refugees to resettle in the U.S. since its inception [54]. However, refugee ceilings can be determined by the executive order, and the quota of refugees allowed into the U.S. is decided each year [5]. For example, the executive order changed the refugee quota of 110,000 allotted in 2016 to 50,000 in 2017 [55]. Similarly, the refugee admittance program became inactive after the US terrorist attacks in 2001, owing to security measures [54].

Muslims comprised 46% of all refugees admitted to the U.S. in 2016 [54]. However, despite this seemingly favorable acceptance of Muslim refugees, recent U.S. polling indicates a contrary populist opinion, with over 50% of U.S. voters not supporting the acceptance of Syrian refugees [54]. While national security remains a legitimate concern, the denial of refugees into the U.S. based on unsupported health information is worrisome. The overarching tenet of medicine and public health is to judiciously draw objective conclusions from scientific evidence to assess disease. Accordingly, physicians, public health practitioners, and refugee health advocates are in the best position to evaluate the merits of immigration policy pertaining to health concerns.

To date, the U.S. State Department, with the advice of the U.S. Department of Justice, has resumed its usual operations of admitting refugees [55,56].
